# Left Ventricular Involvement in Arrhythmogenic Right Ventricular Dysplasia/Cardiomyopathy Predicts Adverse Clinical Outcomes: A Cardiovascular Magnetic Resonance Feature Tracking Study

**DOI:** 10.1038/s41598-019-50535-z

**Published:** 2019-10-02

**Authors:** Meng-ting Shen, Zhi-gang Yang, Kai-yue Diao, Li Jiang, Yi Zhang, Xi Liu, Yue Gao, Bi-yue Hu, Shan Huang, Ying-kun Guo

**Affiliations:** 1 Department of Radiology, State Key Laboratory of Biotherapy, West China Hospital, Sichuan University, Chengdu, China; 2 Department of Radiology, Key Laboratory of Birth Defects and Related Diseases of Women and Children of Ministry of Education, West China Second University Hospital, Sichuan University, Chengdu, China

**Keywords:** Risk factors, Cardiovascular biology

## Abstract

The aim of this study was to investigate left ventricular (LV) global myocardial strain and LV involvement characteristics in patients with arrhythmogenic right ventricular dysplasia/cardiomyopathy (ARVD/C) and to evaluate their predictive value of adverse cardiac events. Sixty consecutive ARVD/C patients with a definite diagnosis of ARVD/C who underwent CMR examination and thirty-four healthy controls were enrolled retrospectively. The CMR images were analyzed for LV myocardial strain and the presence of LV involvement. The endpoint was defined as a composite of sustained ventricular tachycardia or fibrillation, cardiac death, resuscitated cardiac arrest, heart transplantation, and appropriate implantable cardioverter-defibrillator shock. LV global longitudinal (GLS), circumferential (GCS), and radial strain (GRS) were significantly impaired in ARVC/D patients compared to healthy controls (GLS: −13.89 ± 3.26% vs. −16.68 ± 2.74%, GCS: −15.65 ± 3.40% vs. −19.20 ± 2.23%, GRS: 34.57 ± 11.98% vs. 49.92 ± 12.59%; P < 0.001 for all). Even in ARVC/D patients with preserved LVEF, LV GLS, GCS and GRS were also significantly reduced than in controls. During a mean follow-up period of 4.10 ± 1.77 years, the endpoint was reached in 17 patients. LV GLS >−12.65% (HR, 3.58; 95%CI, 1.14 to 11.25; p = 0.029) and history of syncope (HR, 4.99; 95%CI, 1.88 to 13.24; p = 0.001) were the only independent predictors of cardiac outcomes. The LV myocardial deformation derived from FT CMR was significantly impaired in ARVD/C patients, and this alteration can occur before the impairment of LVEF. LV GLS >−12.65% and history of syncope were the only independent prognostic markers of adverse cardiac outcomes.

## Introduction

Arrhythmogenic right ventricular dysplasia/cardiomyopathy (ARVD/C) is a hereditary cardiomyopathy characterized by progressive fat or fibrofatty replacement of the right ventricular (RV) myocardium, which is a common cause of sudden cardiac death (SCD) in the young men and athletes^1^. The cause of ARVD/C is not yet clear, but recent studies suggest it is most often related to desmosomal mutations^2,3^. ARVD/C is not a simple RV disease. Left ventricular (LV) or biventricular involvement are increasingly identified in ARVD/C patients^4^. Cardiac magnetic resonance (CMR) is a valuable diagnostic tool for ARVD/C due to the high negative predictive value, which can identify mild LV impairment and thus has a significant impact on clinical decision-making^5,6^. Prior studies have demonstrated that CMR evidence of LV involvement is a strong independent predictor of hard cardiac events in the entire population, including patients with a definite, borderline, or possible ARVD/C diagnosis^7^. Additionally, the presence of LV dysfunction plays an incremental role in predicting adverse cardiac events compared to RV dysfunction alone^8^.

Myocardial deformation analysis is an objective measurement for the quantification of myocardial function in ARVD/C patients. Previous echocardiographic and CMR studies have determined the use of RV global strain (GS) analysis in the diagnostic work- up of patients with a definite or preclinical ARVD/C^9,10^. Abnormal RV myocardial strain may precede the clinical signs of ARVD/C and also seems to work in ARVD/C family screening protocols^11^. Furthermore, myocardial strain derived from CMR feature tracking (FT) measurements has been shown to be a powerful independent predictor of poor cardiac outcomes in patients with various cardiomyopathies, such as dilated cardiomyopathy or hypertrophic cardiomyopathy^12,13^. To our knowledge, few studies have reported in detail LV global myocardial deformation in ARVD/C patients using CMR FT technology. Moreover, the prognostic role of CMR-based LV GS parameters requires further clarification^11,14^. Thus, the main purpose of this CMR study was to demonstrate LV myocardial deformation and LV involvement characteristics in patients with ARVD/C and to evaluate their predictive value of adverse cardiac events.

## Methods

### Study population

The present study was approved by the Institutional Ethics Review Board of West China Hospital (No. 2016-24) and we pledged to abide by the relevant medical research rules of the Helsinki declaration (2000 EDITION). All enrolled subjects provided written informed consent prior to investigation. Seventy-two consecutive patients with a definite diagnosis of ARVD/C who underwent CMR examination were enrolled retrospectively at a single institution from January 2012 to October 2018. The diagnosis of ARVD/C was based on a set of criteria recommend by the revised Task Force Criteria^15^. All included subjects had to meet two major, one major plus two minor, or four minor diagnostic criteria to be diagnosed with ARVD/C. Idiopathic right ventricular outflow tract tachycardia, which mimic ARVD/C, were excluded carefully, as were alternative causes of RV dilation such as idiopathic dilated cardiomyopathy, tricuspid valve disease, and pulmonary hypertension^16^. We also carefully excluded patients with any history of myocardial infarction, dilated cardiomyopathy, myocarditis and other disease which might cause moderate to severe LV change, both by the clinical information and the CMR images. Patients with poor image quality were also excluded from the study. A control group of 34 age- and gender-matched healthy controls was included. The exclusion criteria for controls included structural heart disease, family history of cardiovascular disease, unexplained arrhythmia, and systemic disease, such as hypertension or diabetes mellitus.

### CMR imaging acquisition

All subjects were assessed on a 3.0- T whole- body MR scanner (MEGNETOM Skyra, Siemens Healthcare, Erlangen, Germany) with a dedicated two-element cardiac-phased array coil. The standard electrocardiographic-gated device and breath-hold technique were used without sedation. For the functional analysis and subsequent myocardial strain measurement, 8–12 continuous CMR cine images were acquired from the base to the apex using a retrospectively-gated, balanced, steady-state free precession (bSSFP) sequence in short-axis slices (TR/TE 39.24/1.43 ms, flip angle 65°, field of view 284 × 284 mm, matrix size 139 × 208 mm, slice thickness 6 mm). The long - axis, 2 - chamber, and 4 - chamber view cine series were acquired using the same sequence as the short-axis. The LGE image was obtained 10–15 min after the injection of contrast agent (repetition time/echo time 763/1.99 ms, inversion time 450 ms, flip angle 20°, thickness 8 mm). All data were acquired during multiple end-inspiratory breath holding.

### CMR imaging analysis

All CMR images were imported to a dedicated software program (CVI42; Circle Cardiovascular Imaging, Inc., Calgary, Canada) for analysis. The images were assessed independently by two experienced investigators (Shen, M. T. with two years and Diao, K. Y. with five years of CMR experience); any discrepancies between the investigators were adjudicated by a third investigator (Jiang, L.). The endocardial and epicardial contours were drawn manually per slice at end-diastolic and end-systolic images by one skilled observer, and the moderator bands and papillary muscles were excluded carefully.

Myocardial strain analysis was performed using an FT algorithm throughout the cardiac cycle. LV endocardial and epicardial borders were manually traced in the end-diastolic phase in the bSSFP short- and long-axis sequences. Subsequently, the software depicted the contours of the border automatically and tracked the motion of the tissue voxels in plane throughout the cardiac cycle. Consequently, we used the reconstructed model to analyze LV global longitudinal peak strain (GLS), radial strain (GRS), circumferential strain (GCS). The definition of each strain parameter was described in previous studies^17^.

T1-weighted black blood sequences combined with and without fat saturation sequences were used to identify fatty infiltration, which appeared as hyperintensity on the T1-weighted imaging but was suppressed out on the fat saturation image correspondingly. LGE was used to detect the presence of fibrosis, which was considered only if hyperintensity of myocardial delayed enhancement was demonstrated on both the short-axis and matching long-axis location.

### Definition of LV involvement on CMR

LV involvement was defined as the presence of one or more of the following CMR signs: LVEDV exceeding the upper limit of normal (defined as 95 mL/m^2^ for men and 90 mL/m^2^ for women), LVEF less than 55%, LV LGE, LV intramyocardial fatty infiltration, and LV wall motion abnormalities (WMAs) including (hypokinesia, akinesia, dyskinesia)^18,19^.

### Follow-up and endpoint

Follow-up was performed in all patients at intervals of six months or longer after the CMR scan. A clinical questionnaire was compiled by an experienced clinical physician during telephone contact or periodic ambulatory visitation. The endpoint was defined as a composite of sustained ventricular tachycardia or fibrillation, cardiac death, resuscitated cardiac arrest, heart transplantation, or appropriate implantable cardioverter-defibrillator (ICD) shock (defined as triggered by life-threatening arrhythmias, including ventricular fibrillation or tachycardia). In case of simultaneous cardiac events per patient, the first event was selected. The clinical performance and conventional CMR parameters were chosen as potential predictors of outcomes, as well as LV deformation parameters.

### Statistical analysis

Continuous variables are expressed as mean value ± standard deviation (SD) or medians with interquartile ranges. The Student t test was employed for comparison of normally distributed data when appropriate. Categorical differences between groups were presented as numbers or percentages and evaluated through Chi-square test or Fisher’s exact text. To assess the role of cardiac magnetic resonance features as predictors of endpoints, the variables were considered for entry into the multivariate Cox regression model using forward selection if p < 0.1 in univariate analysis. Receiver operating curve (ROC) optimized cut-offs of LV GRS, GCS, and GLS were selected for Kaplan-Meier curves based on the highest sensitivity and specificity for predicting the endpoint. Categorical parameters derived from conventional and FT CMR imaging were correlated with clinical cardiac outcomes by Kaplan-Meier survival analysis and tested for significance with a log- rank test. To avoid multicollinearity between different parameters, a variance inflation factor of 5 was set. The diagnostic accuracy of global strain parameters was evaluated for all patients with a diagnosis of ARVD/C patients using ROC analysis. The reproducibility of LV myocardial strain parameters was assessed by the intraclass correlation coefficient (ICC) for each of the intra- and interobserver observations. The value of ICC ≥0.75 was considered excellent, <0.75 and ≥0.40 was moderate, and <0.40 was poor. All data were analyzed through using SPSS statistical software (version 22; SPSS Inc., Chicago, IL, USA) and GraphPad Prism (version 7.1; GraphPad Software, La Jolla, Calif). Two-sided P values of <0.05 was considered to be statistically significant.

## Results

### Baseline characteristics

Of the 72 consecutive patients initially enrolled in the study, 12 (17%) subjects were excluded because of inadequate imaging quality for analysis. Thus, 60 ARVD/C patients (60% were males, mean age was 39 years) and 34 controls were finally included. Nineteen (32%) patients had NYHA functional class of III or IV. The LVEF and RVEF were significantly lower in the ARVD/C patients than in the controls (LVEF: 53.87 ± 10.49% vs. 61.95 ± 5.29%; RVEF: 35.03 ± 13.37% vs. 50.75 ± 11.07%, respectively, both P < 0.001). Fat infiltration was observed in 10 individuals (3 with biventricular infiltration and 7 only in the RV). Of all patients with LGE (n = 34), mid-wall patterns were observed in 14, sub-epicardial in 11, and diffuse in 9 patients. Four patients had a family history of ARVD/C.

### LV involvement on CMR

**Figure 1:**
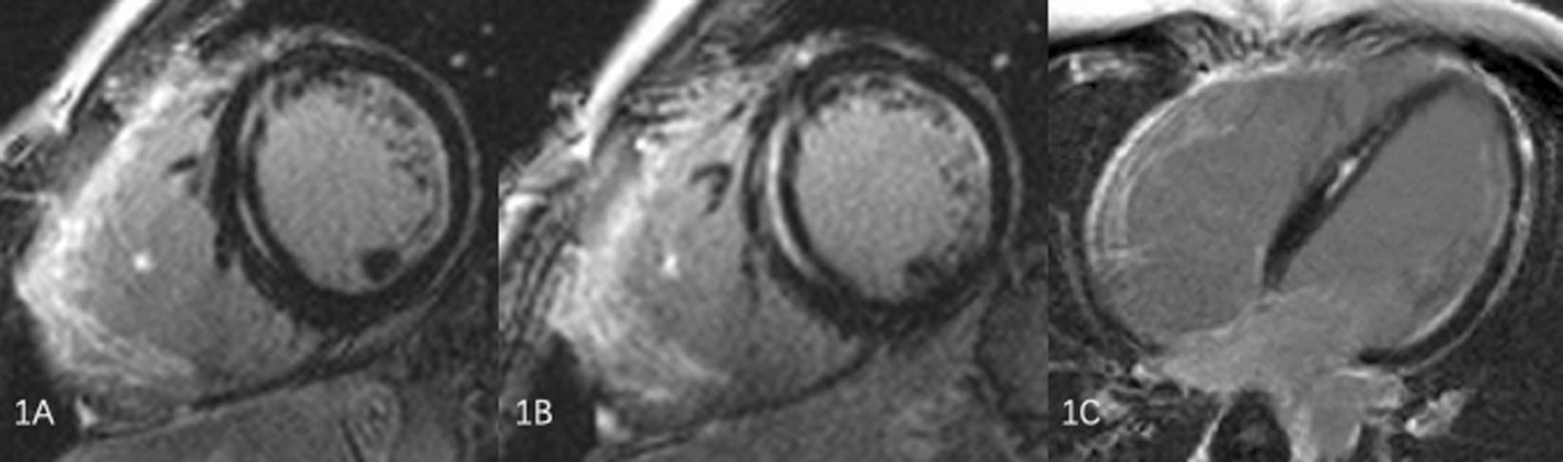
Late gadolinium enhanced at short-axis (**A**,**B**) and long-axis (**C**) CMR image showing a streak of mid myocardial wall delayed enhancement in the septal wall and diffused LGE Of RV wall.

In the current study, 58% (n = 35) of patients had CMR evidence of LV involvement. Within this sub-group, the relevant LV findings included the presence of LV LGE in 23 patients (65%), reduced LVEF in 29 patients (82%), LV dilation in 17 patients (48%), WMAs in 13 patients (37%), and myocardial fat in 3 patients (9%). The dominant distributing locations of LGE were in the mid-myocardial (n = 10) and sub-epicardial (n = 9) (Fig. 1), and the most commonly involved myocardial segments were the posterolateral wall (n = 13). The LVEF for ARVD/C patients with LV involvement was significantly lower than those without such involvement (49.53 ± 10.11% vs. 58.67 ± 9.72%, P = 0.001).

### LV global strain characteristics

ARVD/C patients demonstrated significantly impaired LV GLS, GRS, and GCS compared to healthy controls (P ≤ 0.001 for all). Furthermore, of subgroup ARVC patients with preserved LVEF, the GS was also significantly reduced than in controls (P < 0.001 for all), while no difference was observed in LVEF (59.81 ± 5.49% vs. 61.95 ± 5.29%, p = 0.118) (Table 1). In the sub-group analysis, no difference for LV GS was observed between ARVC patients with and without LV involvement (P = 0.853 for GRS, P = 0.969 for GCS, P = 0.913 for GLS).

**Table 1:** Baseline CMR parameters in ARVC patients and controls.

	Controls (N = 34)	ARVC (N = 60)	ARVC With preserved LVEF (N = 39)
Age (years)	42.32 (13.62)	38.73 (17.65)	38.41 (17.17)
Male, n (%)	20 (58)	36 (60)	25 (64)
**Traditional CMR**			
LVEDV/BSA, ml/m^2^	73.48 (20.31)	72.23 (24.17)	72.19 (24.52)
LVESV/BSA, ml/m^2^	28.26 (10.14)	36.62 (13.90)^‡^	32.17 (9.52)
LVSV/BSA, ml/m^2^	45.11 (11.43)	38.65 (15.30)^‡^	43.00 (14.61)
LVEF, %	61.95 (5.29)	53.87 (10.49)^‡^	59.81 (5.49)
RVEF, %	50.75 (11.07)	35.03 (13.37)^‡^	39.16 (12.64)^§^
Fatty Infiltration, n (%)	0	10 (17)^‡^	4 (10)^§^
LGE, n (%)	0	34 (57)^‡^	19 (48)^§^
**Feature tracking CMR**			
GRS, %	49.92 (12.59)	34.57 (11.98)^‡^	34.67 (12.32)^§^
GCS, %	−19.20 (2.23)	−15.65 (3.40)^‡^	−15.34 (3.26)^§^
GLS, %	−16.68 (2.74)	−13.89 (3.26)^‡^	−14.23 (3.37)^§^

Note: Values are mean (SD) or n (%) as appropriate. ^‡^p < 0.05 vs. normal, ^§^P < 0.05 vs. normal.

CMR, cardiac magnetic resonance; LV, left ventricular; RV, right ventricular; BSA, body surface area; EF, ejection fraction; EDV, end-diastolic volume; ESV, end-systolic volume; SV, stroke volume; LGE, late gadolinium enhancement; GRS, global radial strain; GCS, global circumferential strain; GLS, global longitudinal strain.

**Figure 2:**
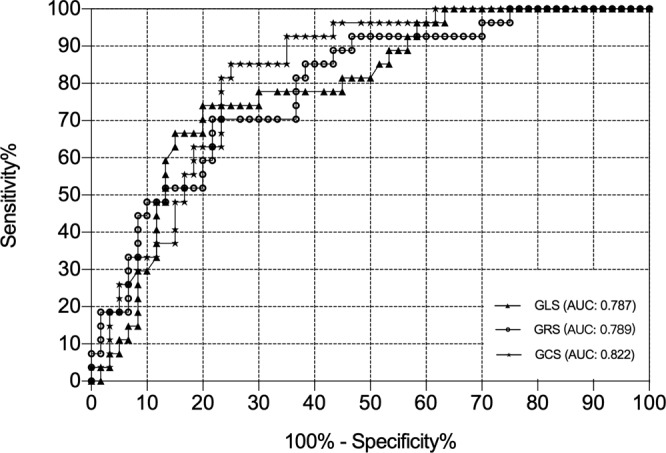
The ROC analysis of three global strains to discriminate ARVD/C patients from controls.

The ROC analysis of all the GS values and LVEF showed a moderate discriminating capacity between ARVD/C patients and controls (AUC of GLS: 0.787, GCS: 0.822, GRS 0.789, P < 0.001 for all; AUC of LVEF: 0.605, p = 0.09) (Fig. 2). Among these parameter, LV GCS displayed the highest sensitivity and specificity with values of 81.48% and 76.67%, respectively, using a cut- off value of less than −17.68% in distinguishing between patients with ARVD/C and controls. The baseline characteristics and other FT CMR parameters of ARVD/C patients and controls were shown in Supplementary Table 1.

### Clinical outcomes

During a mean follow- up period of 4.10 ± 1.77 years, seventeen patients reached the endpoint (some patients experienced more than one event): ten patients reported sustained ventricular tachyarrhythmias, six patients experienced appropriate ICD shock, two patients experienced cardiac arrest, and one patient received a heart transplantation. No deaths occurred during this period. Compared to ARVD/C patients without cardiac events, patients with events had a higher prevalence of syncope and ICD therapy (P ≤ 0.001 for both) and a greater incidence of LGE and LV involvement (P = 0.019 and P = 0.004, respectively). No differences were found for age, sex, or other clinical characteristics. LV GRS, GCS and GLS were significantly lower in patients with cardiac events than in patients without cardiac events (P < 0.05 for all). However, other conventional myocardial function parameters were similar between these two groups (Table 2).

**Table 2:** Baseline characteristics and CMR parameters in ARVC patients with and without adverse cardiac event.

	ARVC/D with events (N = 17)	ARVC/D without events (N = 43)	P value
**Clinical data**			
Follow-up (Y)	3.57 (1.60)	4.16 (1.82)	0.227
Age (Y)	44.12 (19.15)	36.60 (16.78)	0.168
Male, n (%)	11 (65)	25 (58)	0.773
Hypertension, n (%)	2 (18)	2 (6)	0.393
Diabetes, n (%)	0	2 (6)	1.000
History of syncope	9 (53)	2 (5)	**0.000**
ICD	6 (35)	1 (2)	**0**.**001**
Family history of ARVC	2 (12)	2 (5)	0.317
Family history of SCD	0	0	—
NYHA III – IV, n (%)	9 (53)	10 (23)	**0**.**035**
**CMR conditional parameters**			
LVEDV, ml/m^2^	78.40 (21.08)	63.37 (25.17)	0.166
LVESV, ml/m^2^	42.43 (14.85)	34.79 (14.19)	0.074
LVSV, ml/m^2^	39.61 (15.17)	37.73 (15.25)	0.669
LVEF, %	49.57 (12.08)	54.83 (10.11)	0.124
RVEF, %	33.83 (12.11)	35.51 (13.94)	0.647
Fatty infiltration, n (%)	3 (18)	7 (16)	1.000
LGE, n (%)	14 (82)	20 (47)	**0**.**019**
LV involvement on CMR	15 (88)	20 (47)	**0**.**004**
**Feature tacking parameters**			
LV GRS, %	28.53 (9.64)	36.98 (12.06)	**0**.**013**
LV GCS, %	−13.72 (3.48)	−16.42 (3.09)	**0**.**010**
LV GLS, %	−12.07 (3.81)	−14.62 (2.75)	**0**.**005**

Note: Values are mean (SD) or n (%) as appropriate.

Abbreviations: ICD, implantable cardioverter-defibrillator; SCD, sudden cardiac death; NYHA, New York Heart Association; all the other abbreviations are the same as Table 1.

**Figure 3:**
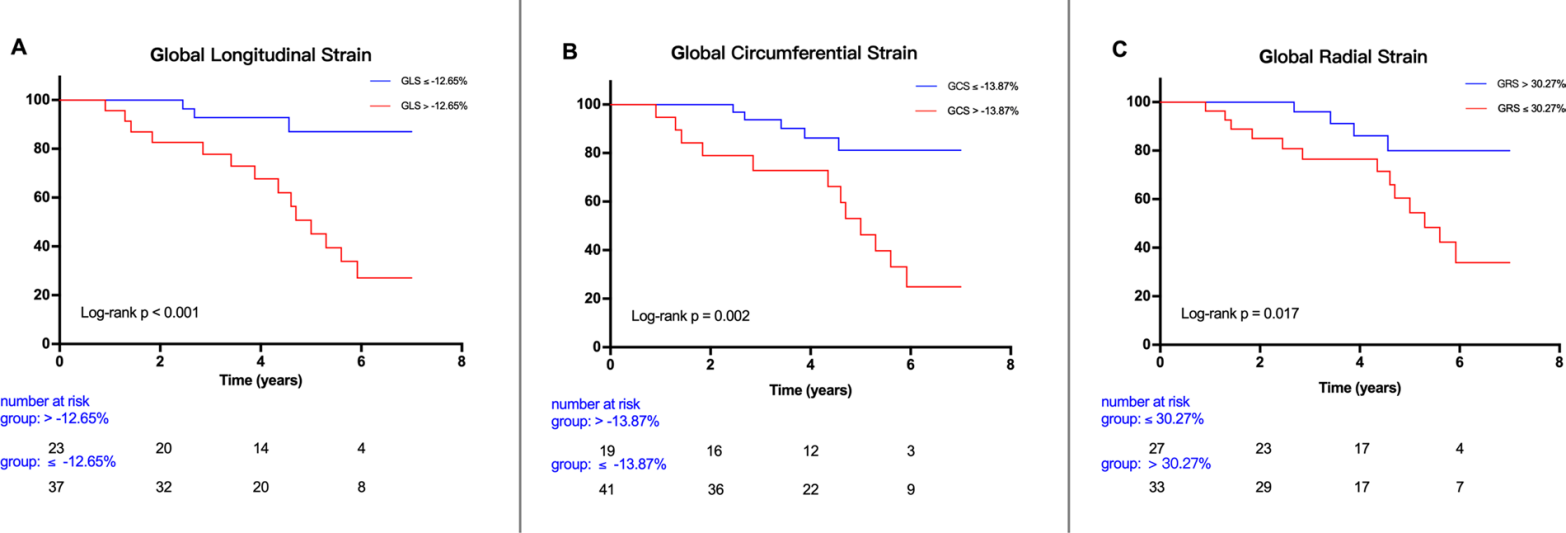
Kaplan-Meier curves for the combined endpoint event-free survival. Patients with global longitudinal strain >−12.65% (**A**), global circumferential strain >−13.38% (**B**), and global radial strain ≤30.27% (**C**) experienced a significantly higher rate of cardiac events.

Kaplan-Meier curves for the endpoint event-free survival demonstrated that patients with LV GLS > −12.65%, LV GCS > −13.87%, and LV GRS <30.27% experienced a significantly higher rate of hard cardiac events (Fig. 3A–C).

In the univariate analysis, several clinical parameters including history of syncope, ICD, LV involvement on CMR, LVEF < 50%, presence of LGE, LV GRS <30.27%, GCS > −13.87%, GLS > −12.65% were univariate predictors of the cardiac endpoint. At first, we tested in multivariate Cox regression models the predictive value of the three direction global strains. In this model, LV GLS > −12.65% (HR 4.8, P < 0.001) remained as independent predictor for cardiac outcomes. Subsequently, a multivariate Cox regression model was built including the mentioned significant univariate variables above. Hereby, LV GLS > −12.65% (HR: 3.58, 95%CI: 1.13 to 11.25, p = 0.029) was significantly associated with cardiac events in addition to the history of syncope (HR: 4.99; 95% CI: 1.88 to 13.24; p = 0.001) after adjusting for age and sex (Table 3).

**Table 3:** Univariate and multivariate Cox proportional hazard models to define the independent impact of on cardiac events.

Variable	univariable analysis			multivariable analysis		
	HR	95% CI	P value	HR	95% CI	P value
Age	1.011	0.983–1.039	0.447			
Sex	1.371	0.506–3.715	0.535			
History of syncope	6.496	2.493–16.924	<0.001	4.99	1.881–13.237	0.001
ICD	6.748	2.469–18.443	<0.001			
NYHA III - IV	0.455	0.175–1.188	0.108			
LV involvement on CMR	5.49	1.255–24.022	0.024			
Presence of LGE	3.577	1.027–12.458	0.045			
Fat infiltration	1.068	0.306–3.731	0.918			
LVEDVi	1.009	0.996–1.022	0.178			
LVESVi	1.01	0.992–1.029	0.289			
LVEF <50%	2.806	1.052–7.487	0.039			
RVEF <40%	0.971	0.337–2.797	0.957			
LV GLS >−12.65%	4.798	1.560–14.755	<0.001	3.578	1.139–11.245	0.029
LV GCS >−13.87%	4.613	1.620–13.140	0.002			
LV GRS <30.27%	2.838	0.997–8.081	0.017			

Abbreviations: ICD: implantable cardioverter-defibrillator, NYHA: New York Heart Association, LV: left ventricular, CMR: cardiac magnetic resonance, LGE: late gadolinium enhancement, LVEVDi: LV end-diastolic volume index, LVESVi: LV end-systolic volume index, LVSV: LV stroke volume, LVEF: left ventricular ejection fraction, RVEF: right ventricular ejection fraction, GLS: global longitudinal strain, GCS: global circumferential strain, GRS: global radial strain.

### Reproducibility

Interobserver and intraobserver ICC were performed in all ARVC patients with values of 0.930 and 0.964 for LV GCS measurements, 0.825 and 0.904 for LV GRS measurements, and 0.812 and 0.897 for LV GLS measurements, respectively.

## Discussion

In this study, we assessed the performance and clinical impact of LV involvement in ARVD/C patients using both conventional and FT- CMR methods and looked at the predictive value of LV strains determined on FT- CMR. We found that LV deformation analysis is superior to LVEF in detecting early LV function change and in differentiating ARVD/C from controls, LV GLS and history of syncope are the independent predictors of cardiac event. These findings have great implication on clinical decision, which might help improve clinical outcomes.

### LV involvement in ARVD/C patients

The present study indicates that most of the ARVD/C patients had CMR evidence of LV involvement, of which LGE and LVEF were the most sensitive indicators. Our study showed that the LV posterolateral wall was the favored site for LGE and primarily occurred in a mid-wall or subepicardial pattern, which is in agreement with previous studies^20,21^. Te Riele *et al*. put forward a new “biventricular triangle,” comprising the LV posterolateral wall, the RV basal inferior wall, and the RV basal anterior wall in limited and moderate mutation-positive ARVD/C patients; they determined that the original “RV apex” was only involved in advanced ARVD/C patients in their study^22^. A subsequent study later confirmed this finding^23^. In the past, LV involvement was frequently considered a late manifestation of advanced disease; however, recent studies at the level of genetic characterization have hypothesized that potential LV involvement may develop ahead of significant RV dysfunction in patients with ARVD/C. The RV dysfunction presents earlier or is more prominently noticed because of its thin and distensible features, which allow it to be primarily affected by the desmosomal mutation and thus present dysfunction^19^.

### LV strain impairment in ARVD/C patients

Myocardial strain analysis has been widely recognized to detect early LV contractility abnormalities^24,25^. In our study, most of the ARVD/C patients had severe LV dysfunction and demonstrated significantly impaired LV global strains than the healthy controls. This result is partly in line with a previous study by Heermann P *et al*., who reported only reduced GLS and GCS rates at the basal level in ARVD/C patients^26^. Although we observed more severe strain impairment, we believe this likely occurred because the population of their study had preserved and higher LV systolic function than our study population (LVEF: 60.7% ± 9.2% vs 53.87 ± 10.49%, respectively). Namely, our participants may be in a more advanced disease stage, which results in greater and more extensive global and regional impairment. Importantly, LV global strain was also impaired in ARVC patients with preserved LVEF compared with controls, even though LVEF did not differ between these two groups. This implies that minor structural LV involvement is already occurred before LV systolic dysfunction exists. Deformation imaging method has greater sensitivity than conventional CMR parameters.

Our study recognized that LV GCS has the highest diagnostic accuracy with a cut-off value of −17.68%, and the best intra- and interobserver reproducibility, which was also in consistent with previously described studies^27^.

### Prognostic role of CMR-based LV strain

Systolic function is generally predictive of outcomes in cardiomyopathy. Myocardial deformation has proved to be a more sensitive method than conventional parameters like ejection fraction in the setting of prognostic aspect. Previous study has confirmed that LV deformation was an independent prognostic marker of outcomes using 2D-STE parameters in ARVD/C patients. In our study, we extend this finding by showing that LV deformation assessed by FT CMR was also an independent predictor for adverse cardiac outcomes in ARVD/C patients^28^. Mast TP et.al showed LV involvement detected by STE (defined as the presence of abnormal systolic peak strain <−12.5% and/or postsystolic shortening >15% in two adjacent LV segments) was an independent predictor of outcomes (HR: 4.9). While the LV involvement using conventional echocardiography (defined as LVEF <50% or the presence of akinesia or dyskinesia) was not the significant predictive marker in multivariate regression. Similarly, in the present study, we find LV GLS assessed by FT CMR was the independent risk factor instead of conventional CMR parameters. Our study observes that 35% of the ARVD/C patients who experienced cardiac events were identified to have no or mild LV involvement. That means adverse events may occurred in ARVD/C patients even in the absence of structural LV alteration. Hence, it is understandable that myocardial strain, compared with conventional CMR parameters, is superior in detecting minor LV involvement and effective in adverse outcomes.

Although there are several possible explanations, the exact mechanism by which myocardial strain predicts adverse cardiac outcomes still remains unclear. In the previous studies, desmosomal mutations are considered to be responsible for the progressive loss of myocytes, and their replacement with fibrofatty tissue. Histopathologic changes can favour re-entry circuits of electrical activation and provide substrates for ventricular arrhythmias. The clinical adverse characteristics of ARVD/C patients may be the result of dual function of the molecular and histopathologic alteration^29^. Researchers believe that LV GLS reflects perturbations in cellular physiology and energetics, and thus dynamically reflects the overall myopathic state and function of the myocardium.

### Prognostic role of syncope

Syncope is a common symptom in ARVD/C patients. It was first identified as an important risk marker in ARVD/C patients in a long-term follow-up by Marcus *et al*. in 1982^30^, and considerable studies describing risk stratifications in patients with ARVD/C were published since then^14,31^. A meta-analysis collected these studies and summarized that syncope was a predictor of ventricular arrhythmias in patients with definite ARVD/C (HR: 3.67, 95%CI:2.75–4.90) and possible ARVD/C (HR: 2.04, 95%CI: 0.39–10.74) respectively^32^. However, the previous studies largely focus on the long-term prognostic role of syncope, our results contributed by indicating that syncope was also a predictor of the endpoint at mid-term follow-up (HR, 4.99; 95%CI, 1.88 to 13.24; p = 0.001). And these findings demonstrated the importance of being alert to the syncope symptom in patients with ARVD/C, as it alerts for adverse cardiac events. Considering recent studies by Orgeron, G. M and Mazzanti, A *et al*. showing that the hazard ratio of syncope identifying ICD therapy for ventricular fibrillation/flutter was range from 1.85 to 3.16^33,34^, and syncope was also significantly associated with the cardiac outcome in ARVD/C patients without ICD^35^. Timing of ICD implantation should be well considered in order to improve clinical efficacy.

### Limitations

Firstly, this was a single-center observational study with a limited number of ARVD/C patients, the cardiac events were retrospectively collected, and we performed both univariate and multivariate cox regression analyses of 17 events that occurred in 60 patients with ARVD/C. Furthermore, twelve patients were excluded from the analysis of LV strain due to suboptimal CMR imaging quality, which may lead to potential limitations in patient selection and event stratification. Secondly, this study collected a cohort of patients who were referred for a CMR scan for the assessment of ARVD/C. From a CMR imaging perspective, the diagnostic sensitivity was decreased according to the revised Task Force Criteria, patients at the preclinical stage of this disease may have been ignored due to mild clinical symptoms. Because no subjects had undergone genetic testing in the present study, potential differences in clinical spectrum and prognostic role of diverse genetic substrates cannot be further ascertained. Additionally, several patients complained of HTN or diabetes, which might but not necessarily result in prominent LV change. Thirdly, the onset of the disease in the single patient is unknown, and the follow-up duration varied by individual patient in our study. Therefore, our evaluation was performed at different stages of the disease in different patients. Additionally, we analysed only clinical performance and CMR data at enrolment. The outcomes can also be influenced by the clinical pharmacotherapy and other therapy. Thereby, an extensive follow-up study should take these into consideration.

## Conclusions

LV global strains assessed by feature tracking cardiovascular magnetic resonance imaging could detect early LV dysfunction and better classify ARVD/C patients. Global longitudinal strain <−12.65% and history of syncope are independent predictors of adverse cardiac events. Routine CMR provides a more sensitive assessment tool to early detect patients who warrant closer clinical follow-up and surveillance, which might help improve clinical outcomes.

## Supplementary information

supplementary table 1

## Data Availability

The datasets used during the current study are available from the corresponding author on reasonable request.
