# Supplementary material for: Left Ventricular Involvement in Arrhythmogenic Right Ventricular Dysplasia/Cardiomyopathy Predicts Adverse Clinical Outcomes: A Cardiovascular Magnetic Resonance Feature Tracking Study
Source: Sci Rep. 2019 Oct 2;9:14235. doi: 10.1038/s41598-019-50535-z (PMC6775112; doi:10.1038/s41598-019-50535-z)
Supplement: Supplementary file 1 — supplementary table 1 [file 41598_2019_50535_MOESM1_ESM.docx]

**Left Ventricular Involvement in Arrhythmogenic Right Ventricular Dysplasia/ Cardiomyopathy**

**Predicts Adverse Clinical Outcomes: A Cardiovascular Magnetic Resonance Feature Tracking Study**

Meng-ting Shen, MS ^1, 2*^; Zhi-gang Yang, MD, PhD ^1†^; Kai-yue Diao, MD ^1*^; Li Jiang, MD ^1^; Yi Zhang, MD ^1^; Xi Liu, MD ^1^; Yue Gao, MS ^1^; Bi-yue Hu, MS ^1^; Shan Huang, MS ^1^; Ying-kun Guo, MD ^2†^

1 Department of Radiology, State Key Laboratory of Biotherapy, West China Hospital, Sichuan University, Chengdu, China.

2 Department of Radiology, Key Laboratory of Birth Defects and Related Diseases of Women and Children of Ministry of Education, West China Second University Hospital, Sichuan University, Chengdu, China.

* These authors contributed equally to this work and should be considered the co-first authors.

† These two authors contributed equally to this work and should be considered corresponding authors.

**Table S1: The baseline characteristics and other FT CMR parameters of all included ARVC patients and controls**

|  | ARVC patients  (N = 60) | Healthy controls  (N = 34) | P value |
| --- | --- | --- | --- |
| Age(years)  Male, n (%)  NYHA III - IV  Hypertension, n (%)  Diabetes, n (%)  Beta blockers, n (%)  ICD implantation  Family history of ARVC  FT-CMR  Radial  TTP, %  PSSR, %  PDSR, %  PD, %  Circumferential  TTP, %  PSSR, %  PDSR, %  PD, %  Longitudinal  TTP, %  PSSR, %  PDSR, %  PD, % | 38.73 (17.65)  36 (60)  19 (32)  4 (7)  2 (3)  6 (10)  7 (12)  4 (7)  424.31 (208.84)  2.01 (1.09)  -2.44 (1.19)  5.47 (1.43)  415.19 (190.84)  -0.82 (0.31)  0.97 (0.39)  0.05 (0.69)  421.16 (191.14)  -0.75 (0.18)  0.80 (0.27)  3.24 (1.48) | 43.32 (13.62)  19 (56)  0  0  0  0  0  0  340.15 (129.05)  2.90 (0.99)  -3.33 (1.16)  6.44 (0.91)  347.63 (131.23)  -1.03 (0.17)  1.24 (0.27)  0.06 (0.12)  368.26 (128.79)  -0.86 (0.14)  1.05 (0.22)  5.81 (2.13) | 0.129  0.828  **< 0.001**  0.293  0.533  0.084  **0.046**  0.293  **0.031**  **< 0.001**  **0.001**  **0.001**  **0.066**  **< 0.001**  **0.001**  0.352  0.146  **0.007**  **< 0.001**  **< 0.001** |

**Note：**Note: Values are mean (SD) or n (%) as appropriate.

**Abbreviations:** TTP, time to peak; PSSR, peak systolic strain rate; PDSR, peak diastolic strain rate; PD, peak displacement. All the other abbreviations are the same as Table 1
